# Respondent Characteristics and Dietary Intake Data Collected Using Web-Based and Traditional Nutrition Surveillance Approaches: Comparison and Usability Study

**DOI:** 10.2196/22759

**Published:** 2021-04-07

**Authors:** Claire M Timon, Janette Walton, Albert Flynn, Eileen R Gibney

**Affiliations:** 1 Centre for eIntegrated Care School of Nursing, Psychotherapy and Community Health Dublin City University Dublin Ireland; 2 Department of Biological Sciences Munster Technological University Cork Ireland; 3 School of Food and Nutritional Sciences University College Cork Cork Ireland; 4 Institute of Food and Health University College Dublin Dublin Ireland

**Keywords:** diet, survey and questionnaire, technology, nutrition surveillance

## Abstract

**Background:**

There are many constraints to conducting national food consumption surveys for national nutrition surveillance, including cost, time, and participant burden. Validated web-based dietary assessment technologies offer a potential solution to many of these constraints.

**Objective:**

This study aims to investigate the feasibility of using a previously validated, web-based, 24-hour recall dietary assessment tool (Foodbook24) for nutrition surveillance by comparing the demographic characteristics and the quality of dietary intake data collected from a web-based cohort of participants in Ireland to those collected from the most recent Irish National Adult Nutrition Survey (NANS).

**Methods:**

Irish adult participants (aged ≥18 years) were recruited to use Foodbook24 (a web-based tool) between March and October 2016. Demographic and dietary intake (assessed by means of 2 nonconsecutive, self-administered, 24-hour recalls) data were collected using Foodbook24. Following the completion of the study, the dietary intake data collected from the web-based study were statistically weighted to represent the age-gender distribution of intakes reported in the NANS (2008-2010) to facilitate the controlled comparison of intake data. The demographic characteristics of the survey respondents were investigated using descriptive statistics. The controlled comparison of weighted mean daily nutrient intake data collected from the Foodbook24 web-based study (329 plausible reporters of a total of 545 reporters) and the mean daily nutrient intake data collected from the NANS (1051 plausible reporters from 1500 reporters) was completed using the Wilcoxon–Mann-Whitney U test in Creme Nutrition software.

**Results:**

Differences between the demographic characteristics of the survey participants across the 2 surveys were observed. Notable differences included a lower proportion of adults aged ≥65 years and a higher proportion of females who participated in the web-based Foodbook24 study relative to the NANS study (*P*<.001). Similar ranges of mean daily intake for the majority of nutrients and food groups were observed (eg, energy [kilocalorie per day] and carbohydrate [gram per day]), although significant differences for some nutrients (eg, riboflavin [mg/10 MJ], *P*<.001 and vitamin B12 [µg/10 MJ], *P*<.001) and food groups were identified. A high proportion of participants (200/425, 47.1%) reported a willingness to continue using Foodbook24 for an additional 6 months.

**Conclusions:**

These findings suggest that by using targeted recruitment strategies in the future to ensure the recruitment of a more representative sample, there is potential for web-based methodologies such as Foodbook24 to be used for nutrition surveillance efforts in Ireland.

## Introduction

Dietary assessment is of paramount importance for the surveillance of public health [1]. Conventional methods include food records (prospective), 24-hour dietary recalls (24HDRs), food frequency questionnaires (FFQs), and diet history methodology (retrospective measures). The selection of the dietary assessment methodology to be used in any given situation is dependent on many factors, including the main objective of the study, the level of detail required, and the resources available [2]. One of the most commonly used methods, the 24-hour multipass dietary recall [3] approach, involves a trained researcher interviewing participants about what they consumed in the previous 24 hours. Techniques such as probing for commonly forgotten items, asking questions about food preparation, and using portion size assessment aids (photos and food models) to assess the amounts consumed are used to prompt accurate recall of dietary intake. Although this method is recommended by the European Food Safety Authority (EFSA) for the collection of dietary intake information [4], the cost and feasibility of this method add challenges. Therefore, many studies opt to collect dietary intake data using FFQs, which are less accurate but require less researcher and participant burden.

Previous research on the use of web-based dietary assessment tools has demonstrated the feasibility of their use in terms of large-scale dietary intake data collection [5-9] and has suggested the potential for the collection of dietary intake information at a lower cost and with less attrition compared with traditional interviewer-led methods [10]. The use of validated, web-based dietary assessment methodologies also allows for estimated intake to (1) be updated frequently through repeated measurements, (2) be investigated across seasons, (3) improve the capture of episodically consumed items, and (4) allow for intraperson and interperson variability to be easily assessed [11]. However, Shim et al [12] noted that even with the use of novel technologies in dietary assessments, the results highlight that participants still have difficulty in reporting diet accurately (underreporting and social desirability bias). A concern is that the data collected using these approaches is flawed with measurement error and, as a result, cannot be confidently relied on to inform public health policy or nutrition- and health-related research [13].

These criticisms are not unique to technology-based self-reported methods but are, in fact, unique to all self-report dietary assessment methodologies, including paper-based measures such as estimated food diaries and interviewer-administered 24HDRs [14]. However, rather than adding to the error, some research demonstrates that technology-based dietary assessment technologies offer a structured data collection approach that reduces the impact of inconsistencies related to erroneous data entry and allows probing into multiple details of the consumption to occur in a harmonized manner and reduce nonresponse bias, as they might be viewed more favorably by participants [15].

National food consumption surveys are necessary to estimate dietary intake at the population level to provide an evidence base for developing and evaluating health policy and to investigate food safety risks, such as contaminant exposure [1]. A recent review of national nutrition surveys conducted in 53 countries of the World Health Organization European region highlights that none of the surveys identified used mobile phones to collect dietary information, whereas Belgian, German, and Portuguese surveys employed electronic interviews; the Spanish Anthropometry, Intake and Energy Balance Study used tablets; and the Norwegian Ungkost and Swedish Riksmaten used a web-based food diary [16]. However, efforts are underway to harmonize the collection of dietary intake data across Europe by further developing a computerized system (GloboDiet) to assist a reviewer in the administration and analysis of 24-hour recalls with participants [17]. In 2017, the third wave for National Diet and Nutrition Survey (NDNS) Rolling Programme in the United Kingdom (2018-2022) has included plans to consider technological dietary assessment approaches; it is hoped that this will highlight the potential of web- and computer-based approaches in national consumption surveys going forward [1].

The feasibility of a self-administered web-based platform to collect nationally representative data in Ireland has yet to be investigated. The collection of nationally representative consumption data incurs a large financial cost, with the most recent National Adult Nutrition Survey (NANS; 2008-2011) in Ireland, costing approximately €5 million (US $5.9 million) to coordinate and execute [18]. Many dietary assessment methods used in nutrition surveillance require highly skilled interviewers, which increases survey costs and thus impacts the frequency of data collection at a national level (on average every 3-10 years depending on the country, except for the United States, where national food consumption data are collected on a yearly basis in the National Health and Nutrition Examination Survey) [19]. Therefore, there is potential for the use of self-administered web-based dietary assessment platforms to assist with the rolling collection of food consumption data at a national level.

Foodbook24 is a self-administered web-based tool that was developed for an Irish adult population and consists of different components that facilitate the collection of dietary intake data without direct interaction with a researcher. The development, validity, and user acceptability of the Foodbook24 tool are described elsewhere [20,21]. Participants were invited to complete dietary assessments using Foodbook24 via email, and a series of email reminders were scheduled to prompt participants to log in and complete each component. Dietary assessment via Foodbook24 can be completed using a range of technology devices, including smartphones, tablet devices, and laptop or desktop computers, thereby providing efficient routes of access to participants and enabling greater and more affordable geographical reach [1].

In this regard, this study aims to investigate the feasibility of using a web-based dietary assessment tool for the purposes of nutrition surveillance in Ireland by (1) comparing the demographic characteristics of participants that sign up to use the web-based Foodbook24 tool relative to the most recent Irish NANS and (2) investigating the quality of dietary intake data collected via the web-based Foodbook24 tool relative to the most recent Irish NANS by means of a controlled comparison.

## Methods

### Foodbook24

The Foodbook24 project was a collaborative research project between the University College Dublin and University College Cork with the aim of developing and validating a web-based dietary assessment tool for the Irish adult population. In brief, the design of the Foodbook24 tool was informed by guidelines issued on the collection of national food consumption data by the EFSA in 2009 [4], interviews with key stakeholder organizations or institutions in Ireland, and an extensive review of the literature concerning web-based dietary assessment platforms [22]. The final proposed design of Foodbook24 was a self-administered web-based tool consisting of different components that facilitate the collection of dietary intake data without direct interaction with a researcher. These components included a screening and consent stage, demographic questionnaire, 2×24-hour multiple-pass dietary recall (administered on nonconsecutive days), and food frequency and food choice questionnaires (FCQs). Foodbook24 was validated in a population of Irish adults by comparing intakes recorded by Foodbook24 against those recorded by a semiweighed food diary and using biological markers of nutrient intake in blood and urine samples [20]. The results of this study demonstrated the validity of Foodbook24 and user acceptability, as Foodbook24 was preferred by participants (80/118, 67.8%) compared with the traditional diary method.

### Foodbook24 Study

**Figure 1:**
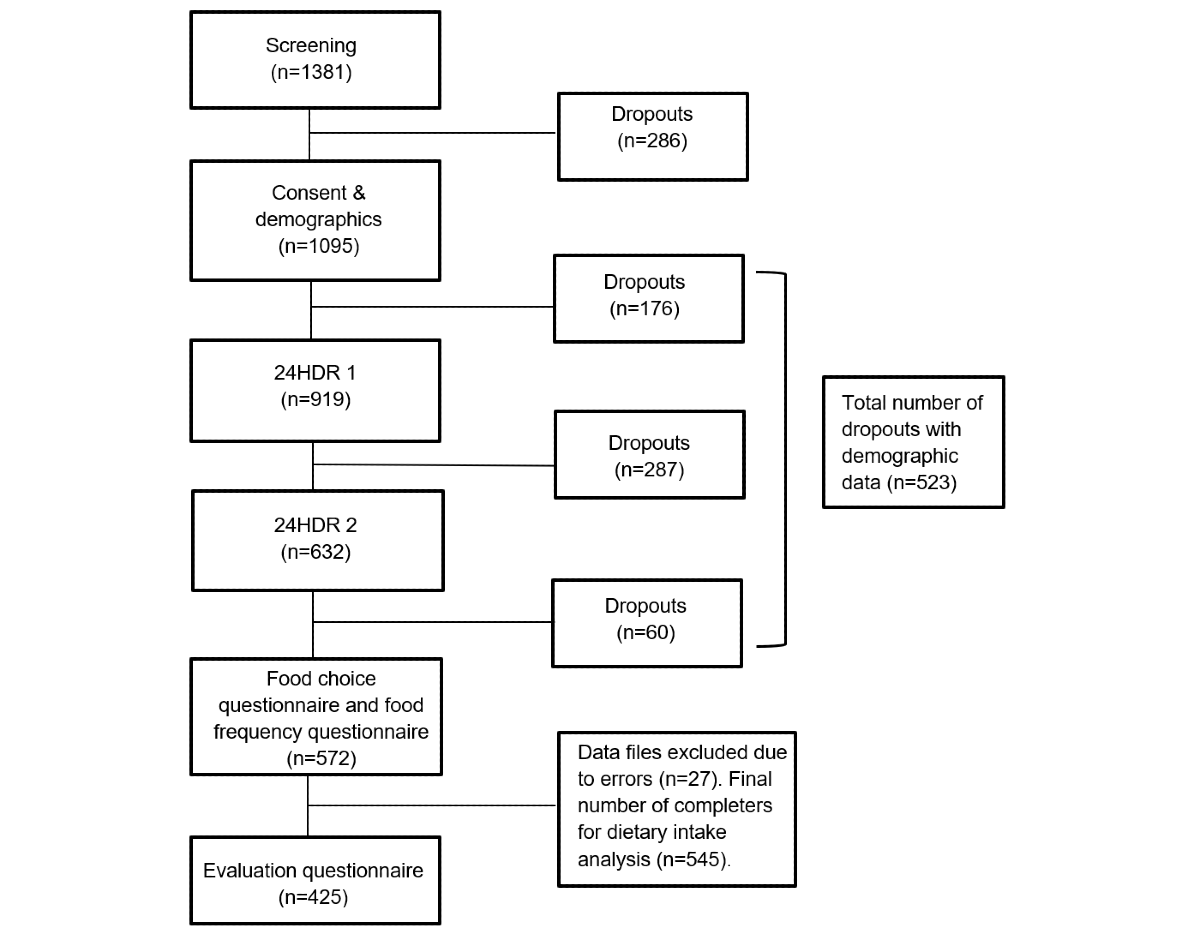
Stages of the Foodbook24 tool in the web-based study. 24HDR: 24-hour dietary recall.

The Foodbook24 study was conducted between March and October 2016. Participants were recruited via the Foodbook24 website, which was aided by advertising of the study in newspapers, posters, e-flyers, social media, and word of mouth. This study was conducted in accordance with the guidelines laid down in the Declaration of Helsinki 1983, and ethical approval was obtained from the University College Dublin Human Research Ethics Committee (LS 15-77 Gibney-Timon). A targeted recruitment strategy to ensure the recruitment of a nationally representative sample of Irish adults (as used in NANS) was not used in this study to allow for the investigation of the demographic characteristics of participants interested in taking part in a study using web-based methodologies. A total of 1385 participants were screened to participate in the web-based study via the Foodbook24 website, and 1095 participants provided demographic data. Participants were eligible to take part in the study if they were aged ≥18 years, fluent in both written and verbal English, had regular access to the internet, and agreed to the information collected as part of the study while ensuring their confidentiality, to be used for the purposes of food and health research. Once participants were screened and provided informed consent using the web-based tool, they had the choice to complete the demographics questionnaire and the first (of two) 24HDR immediately or they had the option to complete these at a later time. A series of email reminders were scheduled to remind participants to log in to the tool and complete the next required component of the tool (Figure 1). The two 24HDRs were separated by a minimum of a 7-day period (may have been longer depending on when participants logged in to complete the second recall), and 2 days after the second recall, the final 2 stages (FFQ and FCQ) were made available for participants to complete; the data of these questionnaires were not included in this publication. Participants who completed all stages of Foodbook24 were asked to complete an evaluation questionnaire once the study had concluded. A total of 425 participants completed the optional questionnaire. The questionnaire consisted of a 16-item evaluation questionnaire administered on the internet. The focus of the questionnaire was to assess the participants’ overall experience using the 24-hour recall component of the tool only and their acceptability of some of the software design features, method preference, and future use. If participants fully complied with the study protocol, study involvement was complete within 10 days. Although financial compensation was not offered for participation in this study, participants who completed all aspects of the study received a personalized dietary feedback report. This report was developed by the research team using the food and nutrient output generated from the tool and further analysis using specialized databases and decision trees that were developed to calculate which food groups contributed the most to nutrient intake. The resulting dietary feedback report was subsequently emailed to the participants.

### National Adult Nutrition Survey (2008-2011)

The NANS investigated habitual food and beverage consumption, lifestyle, health indicators, and attitudes toward food and health in a representative sample (n=1500) of adults aged 18 to 90 years recruited in the Republic of Ireland between 2008 and 2010 [18]. Eligible respondents were adults aged ≥18 years who were free living (living independently in the community) and who were not pregnant or breastfeeding, and a response rate of 60% (1500/2500) was observed. A targeted recruitment strategy was employed in the NANS to ensure representative population samples were recruited. The names and addresses of Irish adults were randomly selected from a database owned by Data Ireland (An Post) to contact potential participants by post. The researchers then contacted potential participants to discuss the study. For groups that were not highly represented via this recruitment strategy, particularly those aged 18 to 35 years, the second level of recruitment was introduced. Analysis of the demographic features in this sample has shown it to be a representative sample of Irish adults with respect to age, gender, social class, and geographical location when compared with census data [18]. Food intake was determined using a 4-day semiweighed food record. At present, this is the most recent nationally representative nutrition survey data available in Ireland.

### Data and Statistical Analysis

#### Nutrient and Food Group Analysis

Food intake data collected from NANS were analyzed using WISP version 4.0 (Tinuviel Software). The food composition data linked to the NANS data set are derived from UK food composition tables [23] and the Irish Food Composition Database [24]. Foodbook24 automatically generates a food and nutrient intake output for each user. The food composition data that underpin the Foodbook24 software were developed via a reduction process that involved the merging of food codes of a similar description and/or composition linked to the NANS data set [25]. Data collected from all participants in the NANS included at least one (of 4) day of dietary intake data recorded on a weekend day, whereas only 31.9% (174/545) of Foodbook24 participants completed one data collection time point on a weekend day.

#### Underreporting

The Henry equation was used to identify misreporters of energy intake (EI) in both surveys. Basal metabolic rate (BMR) was calculated using standard equations based on gender, weight, and age [26]. The mean daily EI and nutrient intake were calculated for all participants in both surveys. In Foodbook24, the nutrient output file was automatically generated, and the data were further aggregated in SPSS (IBM Corporation) to compute the mean daily intakes. Data collected from the NANS were analyzed in WISP to derive mean daily nutrient intake values. Participants whose ratio of EI to their calculated BMR (EI/BMR) fell below 1.1 were classified as underreporters [27], and those with an EI/BMR of >2.5 were classified as overreporters of dietary intake. Individuals identified as misreporters (underreporters and overreporters) were excluded from further analysis, resulting in 329 plausible reporters (of a possible 545 reporters; misreporting rate of 39%) from the Foodbook24 web-based study and 1051 plausible reporters (of a possible 1500 reporters; misreporting rate of 30%) from the NANS.

#### Controlled Comparison of Dietary Intake Data

As there was a large difference in the final number and characteristics of reporters in both surveys, a weighted adjustment was applied to compare population nutrient and food intake recorded in both surveys. Sampling weights were applied to the Foodbook24 data to account for differential probabilities of participant characteristics and nonresponse, applying appropriate sampling weights based on age and gender [28]. The NANS study data were not weighted, as the recruitment strategy ensured a nationally representative sample from the outset. The weighted adjustment and the subsequent modeling of dietary intake collected from the Foodbook24 web-based survey were completed using the Crème Nutrition (R) software.

#### Statistical Analysis

Descriptive statistics (demographic data and evaluation questionnaire data) for both survey populations were computed and compared using a Chi-square analysis in SPSS (version 20). The dietary intake data recorded in both studies were averaged across days, creating mean daily food and nutrient intake, for analysis. The mean, SD, median, and IQR of each nutrient and food group were calculated using Crème Nutrition. The Wilcoxon–Mann-Whitney U test was used to compare the weighted Foodbook24 food and nutrient intake data against intake data recorded from the NANS.

## Results

### Study Populations

A total of 1095 adult participants (766 females and 329 males) signed up to the Foodbook24 web-based study, and 1500 adult participants (740 males and 760 females) were recruited to complete the NANS. As evident from Figure 1, certain stages of the web-based study had higher attrition rates than others, for example, between recall 1 and 2; however, a higher level of adherence was observed for the remainder of the survey after this point. The initial high level of attrition, which can be described as dropout attrition, observed in this study may be partly explained by the fact that for a large number of participants, emails informing them about the next steps required to participate in the study were mistaken as spam mail and therefore were not seen or considered. Table 1 displays the demographic characteristics of the total population of web-based participants compared with those of the NANS participants. The different recruitment approaches resulted in significant differences between the 2 cohorts for all demographic characteristics with notable differences for the representation of the above 65 years age group, male participants, participants who are obese, participants from the manual skilled social class, and participants with a tertiary-level education. Table 1 also shows the demographic characteristics of the Foodbook24 web study completers (those that completed all aspects of the study) and dropouts (those that dropped out without completing all aspects of the study). The analysis demonstrates that a higher proportion of females and participants with a higher level of education completed the study compared with those who dropped out. A subsequent analysis (from Table 2 onward) focuses only on web-based participants who completed 2×24-hour recalls and who were considered adequate energy reporters (n=329) and adequate reporters from the NANS (n=1051).

**Table 1:** Demographic characteristics of participants involved in the Foodbook24 web-based study (2016) and the National Adult Nutrition Survey (2011).

Demographic			Foodbook24 web-based study total population (n=1095)		Foodbook24 web-based study completers (n=572)		Foodbook24 web-based study dropouts (n=523)		National Adult Nutrition Survey (n=1500)		*P* value (difference between Foodbook24 completers and dropouts)			*P* value (difference between 2 surveys)	
Age (years), range			18-89		—^a^		—		18-90		—			—	
**Age (years), n (%)**												.25			<.001^b^
	18-35	501 (45.8)		253 (44.2)		248 (47.4)		531 (35.4)							
	36-50	358 (32.7)		187 (32.7)		171 (33.7)		436 (29.1)							
	51-64	200 (18.3)		110 (17.7)		90 (17.3)		306 (20.4)							
	>65	36 (3.3)		22 (3.9)		14 (2.6)		226 (15.1)							
**Gender, n (%)**												<.001^c^			<.001^b^
	Female	766 (70)		416 (73)		350 (67)		765 (51)							
	Male	329 (30)		156 (27)		173 (33)		735 (49)							
**BMI^d^, n (%)**												.07			<.001^b^
	Underweight (<18.5)	28 (2.6)		16 (2.8)		12 (3.4)		10 (0.7)							
	Normal weight (18.5-24.9)	608 (55.5)		300 (52.4)		300 (57.4)		492 (32.8)							
	Overweight (25-29.9)	331 (30.2)		184 (32.1)		147 (28.1)		532 (35.5)							
	Obese (>30)	127 (11.6)		69 (12.06)		58 (11.1)		318 (21.2)							
**Social class^d^, n (%)**												.24			<.001^b^
	Professional or manager or tech	740 (67.6)		392 (68.5)		348 (66.4)		670 (44.7)							
	Nonmanual skilled	163 (14.9)		83 (14.5)		80 (15.3)		267 (17.8)							
	Manual skilled	22 (2.0)		9 (1.5)		13 (2.5)		213 (14.2)							
	Semiskilled/unskilled	131 (12.4)		59 (10.0)		72 (13.7)		285 (19.0)							
	Retired/unemployed	24 (2.1)		13 (2.2)		11 (1.75)		64 (4.3)							
**Education^d^, n (%)**												.05^c^			<.001^b^
	Primary	16 (1.5)		15 (2.6)		11 (2.1)		139 (9.3)							
	Secondary	188 (17.2)		86 (15.0)		102 (19.6)		650 (43.3)							
	Tertiary	890 (81.3)		480 (84.0)		410 (78.2)		682 (45.5)							

^a^Not available.

^b^Significant difference in demographic information between the Foodbook24 and National Adult Nutrition Survey studies, as defined by Chi-square analysis.

^c^Significant difference in demographic information between the Foodbook24 study completers and dropouts, as defined by Chi-square analysis.

^d^Excludes missing values.

**Table 2:** Nutrient intake of adequate reporters from the Foodbook24 web-based study (2016) and the National Adult Nutrition Survey (2011).

Nutrient	Foodbook24, mean daily intake^a^ (SD)	National Adult Nutrition Survey, mean daily intake^b^ (SD)	*P* value	Difference (%)
Energy (kcal/day)	2174.74 (521.63)	2227.46 (623.56)	.37	2.42
Carbohydrate (g/day)	246.98 (69.98)	252.55 (76.64)	.61	2.25
Starch (g/day)	140.53 (56.24)	146.73 (46.95)	<.001^c^	4.41
Total sugars (g/day)	98.33 (35.6)	101.11 (43.83)	.68	2.83
Dietary fiber (g/day)	24.08 (10.72)	20.67 (8.03)	<.001^c^	−14.17
Fat (g/day)	88.35 (29.75)	84.66 (28.62)	<.001^c^	−4.18
Monounsaturated fat (g/day)	31.26 (11.2)	30.97 (11.25)	.31	−0.93
Polyunsaturated fat (g/day)	14.2 (6.24)	14.8 (6.67)	.16	4.2
Saturated fat (g/day)	36.46 (15.47)	33.35 (12.88)	<.001^c^	−8.53
Protein (g/day)	85.71 (30.51)	89.66 (26.43)	<.001^c^	4.61
Percent energy (protein)	15.83 (3.80)	16.44 (3.41)	<.001^c^	3.67
Percent energy (carbohydrate)	43.17 (8.02)	45.57 (7.29)	<.001^c^	5.27
Percent energy (total sugars)	15.37 (5.83)	18.19 (6.17)	<.001^c^	15.47
Percent energy (fat)	36.52 (7.52)	34.21 (6.30)	<.001^c^	−6.74
Percent energy (monounsaturated fat)	13.07 (3.46)	12.46 (2.67)	.20	−4.93
Percent energy (polyunsaturated fat)	6.11 (2.11)	6.07 (2.29)	<.001^c^	−0.64
Percent energy (saturated fat)	14.71 (4.40)	13.44 (3.44)	<.001^c^	−9.41
Calcium (mg/10 MJ)	1029.46 (356.17)	1124.308 (438.46)	<.001^c^	8.43
Carotene (µg/10 MJ)	5185.62 (4799.66)	4545.71 (3990.98)	.84	−14.07
Copper (mg/10 MJ)	1.42 (0.45)	1.44 (1.83)	.05	1.71
Folate (µg/10 MJ)	322.44 (125.82)	434.35 (326.35)	<.001^c^	25.76
Iron (mg/10 MJ)	14.51 (4.07)	17.21 (19.80)	.42	15.70
Magnesium (mg/10 MJ)	363.79 (87.89)	344.85 (100.62)	<.001^c^	−5.49
Potassium (mg/10 MJ)	462.74 (357.46)	593.44 (829.67)	.66	22.02
Retinol (µg/10 MJ)	1.83 (0.66)	3.76 (9.22)	<.001^c^	51.40
Riboflavin (mg/10 MJ)	2905.23 (940.71)	2923.08 (642.762)	<.001^c^	0.61
Sodium (mg/10 MJ)	2.36 (6.19)	3.57 (9.61)	.06	33.80
Vit B12 (µg/10 MJ)	2.52 (0.90)	4.74 (9.12)	<.01^c^	46.76
Vitamin B6 (mg/10 MJ)	136.64 (93.86)	149.52 (289.00)	<.014^c^	8.61
Vitamin C (mg/10 MJ)	3.24 (2.73)	5.51 (7.53)	<.001^c^	41.24
Vitamin D (µg/10 MJ)	13.09 (5.08)	16.57 (35.40)	<.02^c^	20.96
Vitamin E (mg/10 MJ)	10.71 (3.24)	12.25 (8.10)	<.001^c^	12.53

^a^Mean daily intake of energy and nutrients reported in the Foodbook24 web-based study.

^b^Mean daily intake of energy and nutrients reported in the National Adult Nutrition Survey in Ireland.

^c^Significant difference in the reporting of nutrient intake between the 2 dietary assessment surveys, as defined by the Wilcoxon–Mann-Whitney U test.

### Nutrient Intakes From Adequate Reporters From the Web-Based Foodbook24 Study Compared With Those From the NANS

Table 2 shows nutrient intakes (mean [SD]) for dietary intake data recorded by adequate reporters in the Foodbook24 web-based study (weighted data, n=329) and the NANS (n=1051) study with *P* values from the Wilcoxon–Mann-Whitney U test. Multimedia Appendix 1 displays medians and IQRs of nutrient intakes. Comparable estimates were observed for energy, carbohydrate, polyunsaturated fats, carotene, iron, potassium, and sodium intakes, as highlighted by similar IQRs for intake and no significant difference between intakes. Larger differences were mainly associated with micronutrient intakes, such as retinol, vitamin B12, and vitamin C.

In Tables 3 and 4, the nutrient intakes (mean [SD]) for dietary intake data recorded by adequate reporters in the Foodbook24 web-based study (weighted data) and the NANS studies with *P* values from the Wilcoxon–Mann-Whitney U test are shown for female and male participants, respectively. Multimedia Appendices 2 and 3 display the medians and IQR of nutrient intakes. For female participants, there were no significant differences observed in energy and intake of carbohydrate, polyunsaturated fat, carotene, iron, potassium and sodium recorded in both studies. For male participants, no significant differences were observed in the intake of energy, carbohydrates, starch, monounsaturated and polyunsaturated fats, carotene, iron, magnesium, potassium, retinol, sodium, and vitamin D. Smaller differences in micronutrient intake were observed in male participants from both surveys compared with female participants.

**Table 3:** Nutrient intakes of female adequate reporters from the Foodbook24 web-based study (2016) and the National Adult Nutrition Survey (2011).

Nutrients	Foodbook24, mean daily intake^a^ (SD)	National Adult Nutrition Survey, mean daily intake^b^ (SD)	*P* value	Difference (%)
Energy (kcal/day)	1899.20 (388.60)	1891.82 (425.81)	.25	−0.39
Energy (KJ/day)	7946.26 (1625.93)	7915.38 (1781.59)	.25	−0.39
Carbohydrate (g/day)	218.76 (57.31)	218.39 (55.61)	.63	−0.17
Total sugars (g/day)	80.28 (33.29)	90.28 (34.98)	<.001^c^	11.08
Starch (g/day)	118.32 (39.39)	123.58 (34.09)	<.001^c^	4.26
Protein (g/day)	74.23 (21.75)	75.89 (18.73)	.005^c^	2.19
Fat (g/day)	77.79 (23.54)	73.24 (21.00)	.01^c^	−6.20
Monounsaturated fat (g/day)	27.76 (8.95)	26.56 (8.51)	.02 ^c^	−4.49
Polyunsaturated fat (g/day)	12.94 (4.80)	13.52 (5.61)	.17	4.29
Saturated fat (g/day)	31.64 (12.87)	28.54 (9.58)	<.001^c^	−10.84
Percent energy (protein)	15.71 (3.59)	16.36 (3.31)	<.001^c^	3.95
Percent energy (carbohydrate)	43.34 (8.26)	46.48 (6.57)	<.001^c^	6.74
Percent energy (total sugars)	15.95 (6.11)	19.09 (5.89)	<.001^c^	16.41
Percent energy (fat)	36.67 (7.53)	34.90 (6.06)	<.001^c^	−5.09
Percent energy (monounsaturated fat)	13.15 (3.50)	12.61 (2.60)	<.001^c^	−4.31
Percent energy (polyunsaturated fat)	6.16 (2.05)	6.45 (2.38)	.10	4.55
Percent energy (saturated fat)	14.76 (4.35)	13.61 (3.43)	<.001^c^	−8.48
Dietary fiber (g/day)	22.42 (7.74)	18.99 (7.12)	<.001^c^	−18.06
Calcium (mg/10 MJ)	1039.28 (362.02)	1200.53 (525.56)	<.001^c^	13.43
Carotene (µg/10 MJ)	5539.38 (5076.67)	5345.24 (4511.80)	.63	−3.63
Copper (mg/10 MJ)	1.430 (0.43)	1.58 (2.38)	.03^c^	9.71
Folate (µg/10 MJ)	322.27 (121.01)	453.04 (339.78)	<.001^c^	28.86
Iron (mg/10 MJ)	14.49 (4.03)	18.88 (25.06)	.25	23.23
Magnesium (mg/10 MJ)	366.60 (83.52)	358.21 (120.25)	<.001^c^	−2.34
Potassium (mg/10 MJ)	3737.89 (882.44)	3758.36 (988.83)	.34	0.54
Retinol (µg/10 MJ)	460.97 (363.38)	575.55 (566.87)	.30	19.91
Riboflavin (mg/10 MJ)	1.81 (0.66)	4.46 (11.85)	<.001^c^	59.27
Sodium (mg/10 MJ)	2891.48 (941.44)	2915.64 (626.97)	.09	0.83
Vit B12 (µg/10 MJ)	4.93 (3.07)	10.46 (57.43)	<.001^c^	52.90
Vitamin B6 (mg/10 MJ)	2.46 (0.86)	5.57 (11.99)	<.001^c^	55.80
Vitamin C (mg/10 MJ)	146.27 (96.64)	186.36 (378.23)	<.001^c^	21.51
Vitamin D (µg/10 MJ)	3.21 (2.63)	6.25 (7.56)	<.001^c^	48.61
Vitamin E (mg/10 MJ)	13.10 (4.85)	20.13 (44.56)	<.001^c^	34.92

^a^Mean daily intake of energy and nutrients reported in the Foodbook24 web-based study.

^b^Mean daily intake of energy and nutrients reported in the National Adult Nutrition Survey in Ireland.

^c^Significant difference in the reporting of nutrient intake between the 2 dietary assessment surveys as defined by the Wilcoxon–Mann-Whitney U test.

**Table 4:** Nutrient intakes of male adequate reporters from the Foodbook24 web-based study (2016) and the National Adult Nutrition Survey (2011).

Nutrients	Foodbook24, mean daily intake^a^ (SD)	National Adult Nutrition Survey, mean daily intake^b^ (SD)	*P* value	Difference (%)
Energy (kcal/day)	2497.07 (398.62)	2582.557 (602.03)	.10	3.31
Energy (KJ/day)	10447.76 (1667.85)	10805.42 (2518.92)	.10	3.31
Carbohydrate (g/day)	282.79 (60.18)	286.26 (80.02)	.67	1.21
Total sugars (g/day)	90.55 (33.49)	111.94 (49.12)	.04^c^	19.11
Starch (g/day)	168.40 (53.36)	169.27 (46.99)	.48	0.52
Protein (g/day)	101.28 (31.85)	104.73 (27.72)	.02^c^	3.30
Fat (g/day)	100.49 (27.74)	96.24 (30.50)	.04^c^	−4.41
Monounsaturated fat (g/day)	35.79 (11.34)	35.33 (11.89)	.22	−1.29
Polyunsaturated fat (g/day)	16.55 (7.15)	16.33 (7.48)	.46	−1.38
Saturated fat (g/day)	40.56 (14.64)	38.14 (13.94)	.01^c^	−6.36
Percent energy (protein)	16.20 (4.35)	16.52 (3.51)	<.001^c^	1.92
Percent energy (carbohydrate)	42.64 (7.26)	44.66 (7.86)	<.001^c^	4.53
Percent energy (total sugars)	13.63 (4.47)	17.28 (6.33)	<.001^c^	21.11
Percent energy (fat)	36.07 (7.51)	33.53 (6.46)	<.001^c^	−7.58
Percent energy (monounsaturated fat)	12.83 (3.32)	12.30 (2.72)	<.001^c^	−4.29
Percent energy (polyunsaturated fat)	5.95 (2.28)	5.68 (2.13)	<.001^c^	−4.79
Percent energy (saturated fat)	14.55 (4.59)	13.28 (3.44)	<.001^c^	−9.62
Dietary fiber (g/day)	26.63 (12.70)	22.41 (8.79)	<.001^c^	−18.80
Calcium (mg/10 MJ)	1000.01 (338.42)	1047.35 (309.90)	.003^c^	4.52
Carotene (µg/10 MJ)	4124.35 (3677.76)	3738.54 (3191.90)	.77	−10.32
Copper (mg/10 MJ)	1.40 (0.49)	1.31 (0.99)	.04^c^	−6.96
Folate (µg/10 MJ)	322.93 (140.06)	415.48 (311.39)	<.001^c^	22.28
Iron (mg/10 MJ)	14.56 (4.19)	15.53 (12.18)	.38	6.25
Magnesium (mg/10 MJ)	355.36 (99.96)	331.37 (73.51)	.28	−7.24
Potassium (mg/10 MJ)	3501.05 (799.94)	3443.25 (669.11)	.33	−1.68
Retinol (µg/10 MJ)	468.03 (341.16)	611.51 (1029.34)	.17	23.46
Riboflavin (mg/10 MJ)	1.86 (0.69)	3.05 (5.31)	<.001^c^	39.13
Sodium (mg/10 MJ)	2946.47 (943.10)	2930.59 (658.83)	.57	−0.54
Vit B12 (µg/10 MJ)	4.98 (2.93)	6.27 (6.57)	<.001^c^	20.53
Vitamin B6 (mg/10 MJ)	2.71 (0.99)	3.91 (4.56)	<.001^c^	30.55
Vitamin C (mg/10 MJ)	107.75 (78.65)	112.33 (144.26)	<.001^c^	4.08
Vitamin D (µg/10 MJ)	3.32 (3.01)	4.77 (7.44)	.08	30.39
Vitamin E (mg/10 MJ)	13.08 (5.73)	12.97 (22.14)	<.001^c^	−0.82

^a^Mean daily intake of energy and nutrients reported in the Foodbook24 web-based study.

^b^Mean daily intake of energy and nutrients reported in the National Adult Nutrition Survey in Ireland.

^c^Significant difference in the reporting of nutrient intake between the 2 dietary assessment surveys as defined by the Wilcoxon–Mann-Whitney U test.

### Food Group Intakes From Adequate Reporters From the Web-Based Foodbook24 Study Compared With Those From the NANS

Table 5 displays the daily food group intakes (means, SDs, and percentages of consumers; gram per day) for dietary intake data recorded by adequate reporters in the Foodbook24 web-based (weighted data) and the NANS studies with *P* values from the Wilcoxon–Mann-Whitney U test. Multimedia Appendix 4 displays the medians and IQRs of food group intakes. The results of the analysis demonstrated comparable intake ranges across both surveys; however, for some food groups, there were significant differences in the mean daily food group intake. Intakes of *ready-to-eat breakfast cereals*, *white sliced bread and rolls*, *alcoholic beverages*, *carbonated beverages*, *milk*, *potatoes*, *beef*, and *bacon products* were consumed in significantly less amounts in the Foodbook24 web-based survey than in the NANS. However, an increase in the percentage of consumers for *butter*, *citrus fruits*, *coffee*, *lamb, bacon, and pork dishes*, *nonchocolate confectionery*, *nuts*, *other bread* (eg, *linseed*), *other cereals* (eg, *porridge*), *other fruits* (eg, *kiwis*), and *vegetable and pulse dishes* was evident in the Foodbook24 web-based survey compared with NANS.

**Table 5:** Food group intakes (grams) of adequate reporters from the Foodbook24 web-based study (2016) and the National Adult Nutrition Survey (2011).

Food group			Foodbook24, mean (SD)^a^		Consumers, n (%)^b^		National Adult Nutrition Survey, mean (SD)^c^		Consumers, n (%)^d^		*P* value
**Bread, cereals, rice, and pasta**											
	Other breads (eg, linseed bread)	20.37 (35.65)		128 (39.09)		13.84 (26.9)		374 (35.59)		.06	
	Other breakfast cereals (eg, porridge)	85.64 (113.76)		151 (45.76)		40.72 (84.75)		293 (27.88)		<.001^e^	
	Rice and pasta, flours, grains, and starch	54.27 (86.67)		153 (46.67)		34.95 (53.31)		498 (47.38)		.23	
	Ready-to-eat breakfast cereals	18.11 (28.49)		135 (41.21)		24.53 (30.53)		646 (61.47)		<.001^e^	
	White sliced bread and rolls	15.49 (35.33)		83 (25.15)		55.64 (55.19)		832 (79.16)		<.001^e^	
	Wholemeal and brown bread and rolls	61.6 (69.87)		214 (65.15)		56.23 (56.94)		774 (73.64)		.90	
**Beverages**											
	Alcoholic beverages	174.89 (334.14)		126 (38.18)		356.49 (620.15)		638 (60.7)		<.001^e^	
	Coffees	187.85 (219.41)		181 (55.15)		124.85 (211.12)		490 (46.62)		<.001^e^	
	Teas	424.99 (395.92)		237 (72.12)		465.96 (430.98)		874 (83.16)		.13	
	Water	682.87 (928.29)		219 (66.67)		536.84 (588.48)		857 (81.54)		.72	
	Carbonated beverages	25.27 (90.16)		34 (10.3)		84.44 (167.19)		380 (36.16)		<.001^e^	
	Diet carbonated beverages	30.72 (126.05)		27 (8.18)		22.03 (81.75)		127 (12.08)		.39	
**Dairy**											
	Cheeses	12.47 (20.97)		170 (51.82)		15.06 (19.02)		707 (67.27)		.27	
	Butter (over 80% fat)	11.03 (15.02)		184 (56.06)		4.36 (10.61)		396 (37.68)		<.001^e^	
	Whole milk	27.5 (89.88)		56 (16.97)		116.95 (181.83)		669 (63.65)		<.001^e^	
	Low-fat spreads (under 40% fat)	2.14 (5.36)		53 (16.06)		4.47 (11.18)		296 (28.16)		.01^e^	
	Low-fat, skimmed, and fortified milks	43.66 (121.78)		91 (27.58)		99.76 (151.47)		526 (50.05)		<.001^e^	
	Other milks and milk-based beverages	12.53 (89.42)		26 (7.88		16.08 (56.25)		137 (13.04)		.10	
	Yogurts	30.56 (57.81)		121 (36.67)		33.07 (52.22)		447 (42.53)		.04	
**Fruit and vegetables**											
	Bananas	37.88 (50.93)		145 (43.94)		28.9 (42.62)		485 (46.15)		.10	
	Citrus fruits	26.65 (71.24)		87 (26.36)		15.35 (42.56)		213 (20.27)		.10	
	Green vegetables	13.61 (28.33)		100 (30.3)		13.89 (21.78)		474 (45.1)		<.001^e^	
	Other fruits (berries, apples, etc)	125.42 (126.42)		247 (75.15)		53.74 (78.34)		609 (57.94)		<.001^e^	
	Other vegetables	42.58 (50.17)		224 (68.18)		26.72 (32.17)		745 (70.88)		<.001^e^	
	Vegetable and pulse dishes	29.21 (47.43)		177 (53.94)		20.56 (43.67)		477 (45.39)		<.001^e^	
	Potatoes (boiled/baked/mashed)	55.26 (92.45)		128 (38.79)		79.41 (80.51)		801 (76.21)		<.001^e^	
**Meat, eggs, and fish**											
	Beef and veal	15.01 (34.12)		64 (19.39)		19.41 (31.66)		408 (38.82)		<.001^e^	
	Beef and veal dishes	24.65 (64.85)		56 (16.97)		35.03 (57.67)		373 (35.49)		<.001^e^	
	Bacon and ham	9.93 (24.78)		92 (27.88)		22.46 (25.58)		797 (75.83)		<.001^e^	
	Chicken, turkey, and game	33.41 (64.58)		113 (34.24)		28.76 (37.02)		595 (56.52)		<.001^e^	
	Poultry and game dishes	32.32 (98.08)		73 (22.12)		23.24 (48.99)		274 (26.07)		.53	
	Eggs and egg dishes	33.51 (58.53)		131 (39.7)		17.62 (24.61)		550 (52.33)		.91	
	Fish and fish products	27.39 (50.42)		102 (30.91)		24.98 (36.58)		520 (49.48)		<.001^e^	
	Fish dishes	7.35 (31.48)		23 (6.97)		4.59 (19.54)		77 (7.33)		.83	
	Lamb	3.59 (17.43)		11 (3.33)		5.52 (16.47)		143 (13.61)		.02^e^	
	Lamb, pork, and bacon dishes	7.19 (30.62)		30 (9.09)		5.45 (25.96)		72 (6.85)		.59	
	Meat products	8.77 (29.4)		41 (12.42)		17.62 (29.16)		479 (45.58)		<.001^e^	
	Pork	5.36 (20.96)		20 (6.06)		6.64 (17.69)		179 (17.03)		.01 ^e^	
**Cakes, confectionery, and savory snacks**											
	Cakes, pastries, and buns	20.96 (36.35)		128 (38.79)		20.53 (32.24)		510 (48.53)		.04^e^	
	Biscuits, including crackers	27.89 (44.23)		218 (63.33)		14.13 (20.38)		675 (64.22)		<.001^e^	
	Chocolate confectionery	13.64 (20.55)		166 (50.61)		11.05 (16.74)		554 (52.71)		.89	
	Ice creams	10.16 (22.75)		74 (22.42)		6.79 (15.72)		260 (24.74)		.61	
	Nonchocolate confectionery	5.04 (13)		80 (24.24)		3.94 (11.69)		240 (22.84)		.91	
	Savory snacks	5.27 (13.03)		104 (31.52)		6.73 (12.69)		408 (38.82)		.008^e^	
**Soups, sauces, and miscellaneous**											
	Nuts and seeds; herbs and spices	6.04 (13.24)		139 (42.12)		3.44 (10.3)		263 (25.02)		<.001^e^	
	Soups, sauces, and miscellaneous foods	47.48 (76.06)		244 (74.24)		60.75 (71.79)		920 (87.54)		<.001^e^	

^a^Mean daily intake of food groups in grams per day reported in the Foodbook24 web-based study.

^b^Percentage of participants who reported consuming the respective food group in the Foodbook24 web-based study.

^c^Mean daily intake of food groups in grams per day reported in the National Adult Nutrition Survey in Ireland.

^d^Percentage of participants who reported consuming the respective food groups in the National Adult Nutrition Survey in Ireland.

^e^Significant difference in the reporting of food group intake between the 2 dietary assessment surveys, as defined by the Wilcoxon–Mann-Whitney U test.

### Participant Evaluation of Foodbook24

The main results of the participants’ evaluations (n=425) of Foodbook24 during the web-based study are depicted in Table 6. Most participants were very positive in their evaluation of Foodbook24 with regard to completion time, user-friendliness, and remembering to use the tool. Overall, most found the Foodbook24 system to be user-friendly, with 96.9% (412/425) reporting it easy or *Okay* to use. When asked if participants felt that Foodbook24 changed what they ate and drank, 69.8% (297/425) felt it did not change at all, whereas some (119/425, 28%) felt it changed a little. Participants were asked to use Foodbook24 for longer periods to gain insight into the potential long-term use of the tool. The results highlighted that 36% (153/425) of participants would have continued to use the tool for an additional month (considering the completion of two 24-hour recalls per week), and 47.1% (200/425) of participants reported a willingness to use Foodbook24 for an additional 6 months. A small proportion of participants (34/425, 8%) said they would prefer not to continue using Foodbook24 beyond the web-based study.

**Table 6:** Participant acceptability of Foodbook24 in the web-based study (N=425).

Question posed to participant		Participant responses, n (%)
**Impact of Foodbook24 on diet**		
	Changed a lot	9 (2.1)
	Changed a little	119 (28)
	No change at all	297 (69.8)
**Completion time**		
	Too long	42 (9.8)
	Okay	327 (76.9)
	Short	56 (13.1)
**User-friendliness**		
	Difficult	13 (3)
	Okay	127 (29.8)
	Easy/very easy	285 (67.0)
**Remembering to use Foodbook24**		
	Difficult	21 (4.9)
	Okay	191 (44.9)
	Easy/very easy	213 (50.1)
**Use of Foodbook24 for longer periods**		
	1 week	38 (8.9)
	1 month	153 (36.0)
	6 months	200 (47.1)
	No	34 (8.0)

## Discussion

### Principal Findings

This study addressed the potential of a web-based tool to collect meaningful dietary intake data at a national level by comparing the demographic characteristics and a controlled comparison of dietary intakes between adult participants in the Foodbook24 web-based study and a nationally representative sample of the Irish population NANS study. Overall, our findings suggest key differences in demographic characteristics between survey respondents; however, similar ranges of nutrient and food group data were observed across both studies.

### The Recruitment and Retention of Participants to Web-Based Surveys

The successful recruitment and retention of participants in research studies is essential for optimizing validity [29]. Although a relatively large number of respondents signed up to the web-based Foodbook24 study (n=1095), a retention rate of 58% was observed in the web-based Foodbook24 study from consent to the study to the final stage of data collection (FFQ and FCQ stage; Figure 1). The retention rate in the NANS study was not available; however, the NutriNet Sante study reported a similar rate to that within Foodbook24 at 44%, although the numbers recruited as part of the NutriNet study are more substantial. A study examining the retention rates of women enrolled in nutrition studies noted that the use of email, phone, and text message contact improved retention and highlighted the potential of incentives to optimize retention [29].

It is possible that the demographic characteristic differences observed between the web-based Foodbook24 study and the NANS study are large because of the recruitment efforts undertaken in both studies rather than methods by which the surveys were presented and delivered. For the web-based study, targeted recruitment efforts to ensure the recruitment of a nationally representative sample were not undertaken. This allowed for the investigation of the rate and route of recruitment and characteristics of responders to be examined; that is, were older adults signing up to use Foodbook24 without being directly asked to do so? The findings of this research demonstrate that most participants were female with a higher level of education, suggesting that targeted recruitment strategies are needed when recruiting online nutrition studies and surveys if representative samples are to be achieved.

In contrast, the NANS study employed a multistage, stratified recruitment strategy, and although it was costly, this resulted in the successful recruitment of a sample representative of the Irish adult population. To achieve higher participation rates in web-based nutrition surveillance efforts using Foodbook24 in the future, the use of vast, recurrent multimedia campaigns (television, radio, national/regional newspapers, and billboards) should be considered. This recruitment strategy was employed in the NutriNet Sante study, wherein more than 50,000 participants were successfully recruited to web-based nutrition research [30].

Participants’ evaluation of Foodbook24 in the web-based study highlighted a willingness to use the tool on a long-term basis (Table 6; participants willing to use Foodbook24 for 6 months: 200/425, 47.1%) for dietary data collection, which is a significant finding. In the United States, the results from a study by Thompson et al [10] showed that 70.02% (757/1081) of adult participants (n=1081) preferred the web-based, self-administered Automated Self-Administered 24 hours (ASA24) tool over the interviewer-led Automated Multiple-Pass Method. These results indicate that technology-based dietary methods may encourage users to participate in nutrition surveys [1].

### Demographic Characteristics of Participants in the Web-Based Foodbook24 Study Compared With Those From the NANS Study

The results of the web-based Foodbook24 study compared with the NANS study showed significant differences with respect to the demographic characteristics of the populations recruited. A primary challenge for researchers employing web-based self-report surveys is the ability to engage target populations in the survey, as the method heavily relies on self-selection (referring to when survey participants are allowed to decide whether or not they want to participate in a survey) [31]. The key differences observed included the proportion of those aged ≥65 years, the proportion of males to females recruited, and the distribution of participants in BMI, social class, and education level categories.

There was a significantly lower proportion of older adult participants in the web-based Foodbook24 survey compared with the NANS, although the range of ages of the participants in both studies was very similar, which suggests the potential for the use of Foodbook24 in this population. Ward et al [32] demonstrated the potential use of self-administered, web-based, 24-hour recalls in a population of older adults aged between 60 and 85 years, whereby 67.1% (214/319) completed at least one recall and 47.9% (153/319) completed 2 or more recalls. Ward et al [32] also concluded that further support may be required to obtain multiple recalls in this population, which could be a consideration for Foodbook24 going forward.

Gender is an important determinant of health-risk and health-promoting behaviors [33] and yet research suggests that approximately only 20% of participants in health-related research are male [34]. This finding was apparent in the Foodbook24 web-based study, where only 30.04% (329/1095) of participants who signed up to take part in the study were male. Female participants were found to be more likely to complete all aspects of the Foodbook24 web-based study when the incidence of nonresponse or dropout was investigated. This highlights a clear advantage of a stratified, multistage recruitment approach that was employed in the NANS [15] and other national nutrition surveys such as the NDNS in the United Kingdom, where focused efforts are used to ensure an even proportion of males to females are recruited. Ryan et al [35] noted that there are complex barriers hindering male recruitment to health studies, particularly web-based, and that strategies that involve friends and family to aid recruitment can be successful. The difference in social class and education level observed between the web-based study and NANS is consistent with previous reports, which highlight that individuals with lower education level [36] and social class [37] were less likely to complete web-based surveys, potentially because of computer literacy issues. A higher level of education was also observed in those who completed all aspects of the Foodbook24 web-based study compared with those who dropped out. Kirkpatrick et al [37] recently demonstrated that women with low incomes reported dietary intake data relatively well using ASA24-2016; however, their data were less accurate, relative to women with a higher income. Concentrated efforts to ensure representative samples from all population groups are engaged in future web-based surveys and that training and support are available to those less familiar with technology are warranted [38].

In the Foodbook24 web-based study, a higher proportion (608/1095, 55.52%) of participants reported a BMI within the normal BMI category (18.5-24.9) compared with 32.8% (492/1500) in the NANS study and a lower proportion (127/1095, 11.59%) with obesity compared with 21.2% (318/1500) in the NANS. Web-based anthropometric measurements were self-reported compared with measurements taken by trained researchers in NANS; however, research has shown that self-reported anthropometric data can be reliable when validated against in-person measures [39]. As such, it is difficult to decipher whether the anthropometric data reported as part of the web-based Foodbook24 is actually reflective of the population that took part in the study. However, an element of misreporting is expected, as per previous web-based studies where body measurements are self-reported [40].

### Comparison of Dietary Intake Data Collected From the Web-Based Foodbook24 Study and NANS

Although both web-based and interviewer-administered dietary assessment tools are prone to similar measurement errors and correlated person-specific biases [41], research has shown that 24-hour recall tools can provide comparable data to interviewer-administered recalls [10,21,42,43] and are substantially better than FFQs [44,45]. Web-based methodologies for the purposes of nutrition surveillance also provide automated analysis and standardized approaches for the collection of data, which reduces the likelihood of error associated with human data collection and analysis [1].

In this study, the discrepancies observed between intakes from both NANS and the web-based study may be because of different time points of data collection and the changes in food consumption trends between those time points; however, it is important to consider the impact of the different dietary assessment methodologies on nutrient and food group data from both surveys. It is also possible that by presenting the participant with a limited food and beverage list in the Foodbook24 tool compared with open-ended entry options as per the food diary method may also explain some of the discrepancies observed. Future development research to address this potential issue is currently underway. De Keyzer et al [46] compared data collected from repeated 24-hour recalls using EPIC-SOFT, a European computer program for 24-hour dietary protocols, to data collected from a 5-day estimated food diary for estimating nutrient intakes in a national food consumption survey. The results highlighted a similar level of misreporting using both methods, and similar to this study, group-level intakes of protein, carbohydrates, starch, sugar, water, potassium, and calcium from duplicate 24HDRs did not differ from those obtained by 5-day estimated diet records. However, for micronutrients that are concentrated in fewer food items such as vitamin A, more repeated 24-hour recalls are necessary to obtain representative estimates of absolute usual intakes [47].

The data collected from the web-based study compared with the NANS study clearly highlight the potential of Foodbook24 for the rapid identification of food trends over time if used in a rolling data collection capacity. Higher consumption rates of coffee, pulses, and exotic fruits and lower consumption of food items such as white bread and ready-to-eat breakfast cereals were observed in the web-based data compared with NANS. Alcoholic beverages were reported as consumed less frequently in the web-based study compared with NANS, which is likely because of the fact that alcoholic beverages are more frequently consumed on weekend days [48] and data collection on weekend days only occurred in 31.9% (174/545) of web-based participants compared with 100% (1500/1500) of NANS participants.

Estimating the usual intake of episodically consumed foods based on a limited number of 24HDRs per participant can be challenging for their use in national consumption surveys [49] and is an important consideration for using Foodbook24 in large-scale surveys going forward. Potential strategies to address these issues include the use of repeat, preferably nonconsecutive dietary recalls, concurrent blended/combined dietary assessment tools alongside the application of sophisticated statistical modeling, and the collection of biological samples to assess biomarkers of nutrient and food group intake as an independent measure [50].

### Limitations

This study acknowledges the limitations to this analysis, as it was performed using dietary intake data collected using 2 different methodologies (2×nonconsecutive 24-hour recalls vs 4-day semiweighed food diary) at 2 different time points (5 years apart; the Irish NANS was completed in 2011 and the web-based Foodbook24 study was completed in 2016) and in separate adult cohorts (a random adult sample population vs a representative adult population). As such, the differences observed in this analysis may be because of differences in the education, BMI, and social classes of participants involved in the 2 studies, making it inherently difficult to compare. However, a number of efforts have been made to address these limitations, including (1) completing a controlled comparison of dietary intakes by applying sampling weights to the Foodbook24 data to account for differential probabilities of participant characteristics and nonresponse (based on age and gender), and (2) coding and analyzing the data from both cohorts by using the same food grouping structure and compositional food tables to explore the potential of using a web-based platform to collect dietary intake data of a similar quality relative to data collected using a pen- and paper-based dietary assessment method in Ireland.

### Future Considerations for Web-Based Methodologies in Nutrition Surveillance

As it stands, open-source web-based surveys delivered via Foodbook24 do not result in the collection of dietary intake data from a representative sample of the Irish adult population. Although web-based methodologies offer standardized collection and analysis of data, the use of these tools to collect data from representative samples of populations is challenging. Future investigations of the comparison of methodologies should also control for factors such as social class or education, as the findings from this analysis demonstrate that responders with lower socioeconomic status and education were not proportionally represented in the web-based study sample. Although further work is warranted, a carefully designed recruitment strategy for the use of Foodbook24 in national nutrition surveys, especially considering population groups that may require extra support and training, has the potential to exceed the recruitment rates of previous national surveys. Platform adaptations, such as the collection of brand-level data and adapted approaches for groups such as older adults and infants, need to be considered for the collection of nationally representative food consumption information. This research demonstrates the capability of Foodbook24 to collect acceptable food and nutrient intake data from large survey populations. These findings support the use of Foodbook24 as a semicontinuous monitoring system in Ireland that would provide a cost-effective platform to collect valuable information to regularly evaluate the dietary intake of the general Irish adult population. This would allow for the rapid identification of food trends and for the development and monitoring of effective policies on nutrition and food safety in the future.

## Appendix

Multimedia Appendix 1 Nutrient intakes of adequate reporters from the Foodbook24 web-based study (2016) and the National Adult Nutrition Survey (2011).

Multimedia Appendix 2 Nutrient intakes of female adequate reporters from the Foodbook24 web-based study (2016) and the National Adult Nutrition Survey (2011).

Multimedia Appendix 3 Nutrient intakes of male adequate reporters from the Foodbook24 web-based study (2016) and the National Adult Nutrition Survey (2011).

Multimedia Appendix 4 Food group intakes (grams) of adequate reporters from the Foodbook24 web-based study (2016) and the National Adult Nutrition Survey (2011).

## Abbreviations

24HDR: 24-hour dietary recall
ASA24: Automated Self-Administered 24 hours
BMR: basal metabolic rate
EFSA: European Food Safety Authority
EI: energy intake
FCQ: food choice questionnaire
FFQ: food frequency questionnaire
NANS: National Adult Nutrition Survey
NDNS: National Diet and Nutrition Survey
