# Supplementary material for: Respondent Characteristics and Dietary Intake Data Collected Using Web-Based and Traditional Nutrition Surveillance Approaches: Comparison and Usability Study
Source: JMIR Public Health Surveill. 2021 Apr 7;7(4):e22759. doi: 10.2196/22759 (PMC8060863; doi:10.2196/22759)
Supplement: Multimedia Appendix 1 [file publichealth_v7i4e22759_app1.docx]

| **Nutrient** | **FB24** **Median^f^** | **FB24 IQR^g^** | **NANS Median^h^** | **NANS IQR^i^** |
| --- | --- | --- | --- | --- |
| **Energy (kcal/day)** | 2132.07 | (1781.91-2542.52) | 2147.83 | (1792.11-2602.1) |
| **Carbohydrate (g/day)** | 237.24 | (198.92-290.51) | 242.37 | (197.98-299.54) |
| **Starch (g/day)** | 136.24 | (102.32-169.18) | 141.08 | (114.56-173.93) |
| **Total Sugars (g/day)** | 94.73 | (73.50-118.73) | 96.21 | (69.60-125.96) |
| **Dietary Fibre (g/day)** | 22.01 | (17.59-28.07) | 19.36 | (15.10-24.76) |
| **Fat (g/day)** | 86.28 | (68.24-109.55) | 80.93 | (64.18-100.36) |
| **Mono fat (g/day)** | 30.17 | (23.00-38.76) | 29.71 | (22.82-37.28) |
| **Poly fat (g/day)** | 12.98 | (9.92-17.33) | 13.68 | (10.42-17.95) |
| **Sat fat (g/day)** | 34.5 | (25.66-46.81) | 31.66 | (24.59-39.91) |
| **Protein (g/day)** | 81.84 | (65.36-98.72) | 85.83 | (71.72-105.10) |
| **Percent Energy Protein** | 15.24 | (13.27-17.55) | 16.05 | (14.27-18.03) |
| **Percent Energy Carbohydrate** | 43.83 | (38.55-48.25) | 45.59 | (41.24-50.32) |
| **Percent Energy Total Sugars** | 14.54 | (11.50-18.63) | 18.06 | (13.92-22.2) |
| **Percent Energy Fat** | 36.69 | (31.63-40.67) | 34 | (30.22-37.78) |
| **Percent Energy Mono fat** | 12.865 | (11.08-14.91) | 12.32 | (10.7-14.01) |
| **Percent Energy Poly fat** | 5.97 | (4.71-6.99) | 5.76 | (4.54-7.14) |
| **Percent Energy Sat fat** | 14.385 | (11.95-17.41) | 13.25 | (11.2-15.33) |
| **Calcium (mg/10MJ)** | 965.06 | (785.03-1210.07) | 1049.33 | (842-1286.62) |
| **Carotene (µg/10MJ)** | 3984.34 | (1326.60-7791.18) | 3344.95 | (1580.04-6494.81) |
| **Copper (mg/10MJ)** | 1.35 | (1.11-1.65) | 1.2 | (1-1.46) |
| **Folate (µg/10MJ)** | 302.8 | (242.50-394.13) | 371.26 | (285.29-500.32) |
| **Iron (mg/10MJ)** | 13.99 | (11.89-16.30) | 13.85 | (11.63-16.96) |
| **Magnesium (mg/10MJ)** | 350.56 | (301.23-421.80) | 333.42 | (282.16-385.43) |
| **Potassium (mg/10MJ)** | 380.39 | (263.45-568.87) | 405.87 | (271.03-648) |
| **Retinol (µg/10MJ)** | 1.695 | (1.40-2.13) | 2.26 | (1.74-2.94) |
| **Riboflavin (mg/10MJ)** | 2797.78 | (2199.84-3452.24) | 2874.38 | (2464.8-3293.8) |
| **Sodium (mg/10MJ)** | 1.725 | (1.45-2.19) | 1.95 | (1.57-2.55) |
| **Vit B12 (µg/10MJ)** | 2.38 | (1.89-3.00) | 3.1 | (2.45-4.14) |
| **Vitamin B6 (mg/10MJ)** | 114.84 | (68.35-186.93) | 90.36 | (53.04-150.53) |
| **Vitamin C (mg/10MJ)** | 2.64 | (1.38-4.01) | 3.48 | (1.95-6.3) |
| **Vitamin D (µg/10MJ)** | 12.13 | (9.85-15.31) | 11.15 | (8.4-15.61) |
| **Vitamin E (mg/10MJ)** | 10.19 | (8.44-12.32) | 10.82 | (8.96-13) |

Multimedia Appendix 1: Nutrient intakes of adequate reporters from the Foodbook24 Web-based study (2016) and the National Adult Nutrition Survey (2011)

f Median intake of energy and nutrients reported in the Foodbook24 Web-based study

g Interquartile range (IQR) of daily energy and nutrient intakes reported in the Foodbook24 Web-based survey

h Median intake of energy and nutrients reported in the National Adult Nutrition Survey in Ireland

i Interquartile range (IQR) of daily energy and nutrient intakes reported in the National Adult Nutrition Survey in Ireland.
