# Supplementary material for: Respondent Characteristics and Dietary Intake Data Collected Using Web-Based and Traditional Nutrition Surveillance Approaches: Comparison and Usability Study
Source: JMIR Public Health Surveill. 2021 Apr 7;7(4):e22759. doi: 10.2196/22759 (PMC8060863; doi:10.2196/22759)
Supplement: Multimedia Appendix 2 [file publichealth_v7i4e22759_app2.docx]

Multimedia Appendix 2: Nutrient intakes of female adequate reporters from the Foodbook24 Web-based study (2016) and the National Adult Nutrition Survey (2011)

| **Nutrients** | **FB24Median^f^** | **FB24 IQR^g^** | **NANS Median^h^** | **NANS IQR^i^** |
| --- | --- | --- | --- | --- |
| **Energy (kcal/day)** | 1793.95 | (1633.35-2062.62) | 1881.67 | (1633.33-2098.98) |
| **Energy (KJ/ day)** | 7505.88 | (6833.94-8630.01) | 7872.92 | (6833.88-8782.15) |
| **Carbohydrate (g/day)** | 214.72 | (179.26-251.53) | 213.63 | (183.42-250.25) |
| **Total Sugars (g/day)** | 75.21 | (56.72-100.25) | 87.51 | (66.35-112.87) |
| **Starch (g/day)** | 117.53 | (90.22-144.28) | 122.54 | (102.13-143.02) |
| **Protein (g/day)** | 68.86 | (60.13-84.36) | 74.86 | (63.35-86.37) |
| **Fat (g/day)** | 74.26 | (63.43-89.21) | 72.01 | (60.00-86.05) |
| **Mono fat (g/day)** | 26.74 | (21.98-32.94) | 25.31 | (20.94-31.82) |
| **Poly fat (g/day)** | 12.21 | (9.54-15.91) | 12.56 | (9.65-16.63) |
| **Sat fat (g/day)** | 29.45 | (23.54-38.37) | 28.19 | (21.69-33.99) |
| **Percent Energy Protein** | 15.12 | (13.10-17.91) | 15.98 | (14.31-18.06) |
| **Percent Energy Carbohydrate** | 44.01 | (38.78-48.59) | 46.25 | (42.45-50.52) |
| **Percent Energy Total Sugars** | 15.10 | (11.97-19.45) | 19.00 | (15.05-22.78) |
| **Percent Energy Fat** | 36.69 | (31.78-40.75) | 34.50 | (30.86-38.63) |
| **Percent Energy Mono fat** | 12.87 | (11.12-14.90) | 12.39 | (10.87-14.23) |
| **Percent Energy Poly fat** | 6.01 | (4.8-7.02) | 6.21 | (4.82-7.58) |
| **Percent Energy Sat fat** | 14.45 | (12.24-17.42) | 13.53 | (11.38-15.51) |
| **Dietary Fibre (g/day)** | 21.52 | (17.06-26.14) | 18.08 | (14.26-22.26) |
| **Calcium (mg/10MJ)** | 970.32 | (783.3-1234.31) | 1088.61 | (864.10-1358.71) |
| **Carotene (µg/10MJ)** | 4059.73 | (1567.82-8103.51) | 4221.21 | (2022.63-7513.36) |
| **Copper (mg/10MJ)** | 1.41 | (1.10-1.69) | 1.26 | (1.06-1.52) |
| **Folate (µg/10MJ)** | 306.06 | (241.85-398.34) | 380.00 | (285.88-525.32) |
| **Iron (mg/10MJ)** | 14.17 | (11.89-16.26) | 14.09 | (11.89-17.12) |
| **Magnesium (mg/10MJ)** | 360.17 | (303.96-430.23) | 343.27 | (284.81-404.28) |
| **Potassium (mg/10MJ)** | 3715.12 | (3031.00-4340.87) | 3639.10 | (3176.59-4201.55) |
| **Retinol (µg/10MJ)** | 382.91 | (259.59-567.65) | 414.14 | (281.04-648.21) |
| **Riboflavin (mg/10MJ)** | 1.70 | (1.40-2.17) | 2.31 | (1.72-3.18) |
| **Sodium (mg/10MJ)** | 2786.81 | (2149.25-3436.91) | 2886.85 | (2463.71-3262.68) |
| **Vit B12 (µg/10MJ)** | 4.30 | (2.82-6.32) | 5.06 | (3.68-7.37) |
| **Vitamin B6 (mg/10MJ)** | 2.36 | (1.86-2.97) | 3.14 | (2.4-4.17) |
| **Vitamin C (mg/10MJ)** | 132.35 | (73.39-199.85) | 109.75 | (65.64-166.14) |
| **Vitamin D (µg/10MJ)** | 2.71 | (1.40-3.98) | 3.80 | (2.08-7.06) |
| **Vitamin E (mg/10MJ)** | 12.18 | (9.98-15.31) | 12.35 | (9.41-17.09) |

f Median intake of energy and nutrients reported in the Foodbook24 Web-based study

g Interquartile range (IQR) of daily energy and nutrient intakes reported in the Foodbook24 Web-based survey

h Median intake of energy and nutrients reported in the National Adult Nutrition Survey in Ireland

i Interquartile range (IQR) of daily energy and nutrient intakes reported in the National Adult Nutrition Survey in Ireland.
