# Supplementary material for: Respondent Characteristics and Dietary Intake Data Collected Using Web-Based and Traditional Nutrition Surveillance Approaches: Comparison and Usability Study
Source: JMIR Public Health Surveill. 2021 Apr 7;7(4):e22759. doi: 10.2196/22759 (PMC8060863; doi:10.2196/22759)
Supplement: Multimedia Appendix 3 [file publichealth_v7i4e22759_app3.docx]

Multimedia Appendix 3: Nutrient intakes of male adequate reporters from the Foodbook24 Web-based study (2016) and the National Adult Nutrition Survey (2011)

| **Nutrients** | **FB24 Median^f^** | **FB24 IQR^g^** | **NANS Median^h^** | **NANS IQR^i^** |
| --- | --- | --- | --- | --- |
| **Energy (kcal/day)** | 2482.25 | (2206.68-2769.98) | 2550.13 | (2196.36-2940.01) |
| **Energy (KJ/ day)** | 10385.75 | (9232.77-11589.6) | 10669.73 | (9189.6-12301.02) |
| **Carbohydrate (g/day)** | 279.66 | (235.97-325.01) | 283.54 | (233.77-335) |
| **Total Sugars (g/day)** | 86.87 | (65.33-109.32) | 108.63 | (76.84-141.93) |
| **Starch (g/day)** | 155.58 | (129.77-206.91) | 166.98 | (138.88-198.13) |
| **Protein (g/day)** | 93.00 | (80.48-117.46) | 103.40 | (85.68-120.21) |
| **Fat (g/day)** | 100.05 | (78.12-117.02) | 92.92 | (76.53-113.31) |
| **Mono fat (g/day)** | 34.91 | (27.90-43.17) | 34.50 | (27.81-41.18) |
| **Poly fat (g/day)** | 15.51 | (11.87-19.37) | 15.18 | (11.32-19.73) |
| **Sat fat (g/day)** | 39.06 | (31.24-51.90) | 36.76 | (29.03-46.53) |
| **Percent Energy Protein** | 15.33 | (13.47-17.21) | 16.15 | (14.26-18.02) |
| **Percent Energy Carbohydrate** | 42.33 | (37.92-46.96) | 44.86 | (39.77-50.16) |
| **Percent Energy Total Sugars** | 13.35 | (10.39-16.57) | 16.96 | (13.21-21.57) |
| **Percent Energy Fat** | 36.47 | (31.41-40.50) | 33.37 | (29.49-37) |
| **Percent Energy Mono fat** | 12.84 | (11.05-14.93) | 12.25 | (10.5-13.83) |
| **Percent Energy Poly fat** | 5.85 | (4.44-6.94) | 5.47 | (4.25-6.61) |
| **Percent Energy Sat fat** | 14.32 | (11.50-17.11) | 13.07 | (11.04-15.18) |
| **Dietary Fibre (g/day)** | 23.60 | (19.73-30.84) | 21.18 | (16.32-27.39) |
| **Calcium (mg/10MJ)** | 926.96 | (792.66-1106.84) | 989.67 | (827.04-1236.19) |
| **Carotene (µg/10MJ)** | 3444.28 | (881.39-6882.33) | 2818.64 | (1284.08-5447.31) |
| **Copper (mg/10MJ)** | 1.30 | (1.09-1.54) | 1.13 | (0.94-1.37) |
| **Folate (µg/10MJ)** | 296.53 | (242.87-370.54) | 365.92 | (283.1-481.98) |
| **Iron (mg/10MJ)** | 13.56 | (11.86-16.47) | 13.57 | (11.27-16.63) |
| **Magnesium (mg/10MJ)** | 341.25 | (294.30-391.13) | 325.32 | (280.31-371.47) |
| **Potassium (mg/10MJ)** | 3437.37 | (2929.84-3926.27) | 3410.82 | (2978.84-3818.26) |
| **Retinol (µg/10MJ)** | 371.51 | (281.87-572.55) | 398.83 | (264.32-645.22) |
| **Riboflavin (mg/10MJ)** | 1.68 | (1.38-2.03) | 2.24 | (1.76-2.84) |
| **Sodium (mg/10MJ)** | 2822.04 | (2307.21-3560.60) | 2843.08 | (2464.8-3331.23) |
| **Vit B12 (µg/10MJ)** | 4.16 | (2.97-7.35) | 4.95 | (3.54-6.82) |
| **Vitamin B6 (mg/10MJ)** | 2.63 | (1.96-3.22) | 3.07 | (2.51-4.1) |
| **Vitamin C (mg/10MJ)** | 84.09 | (52.80-142.13) | 74.53 | (43.04-128.27) |
| **Vitamin D (µg/10MJ)** | 2.51 | (1.22-4.21) | 3.07 | (1.89-5.72) |
| **Vitamin E (mg/10MJ)** | 11.99 | (9.45-15.81) | 10.10 | (7.23-14.2) |

f Median intake of energy and nutrients reported in the Foodbook24 Web-based study

g Interquartile range (IQR) of daily energy and nutrient intakes reported in the Foodbook24 Web-based survey

h Median intake of energy and nutrients reported in the National Adult Nutrition Survey in Ireland

i Interquartile range (IQR) of daily energy and nutrient intakes reported in the National Adult Nutrition Survey in Ireland.
