# Supplementary material for: Respondent Characteristics and Dietary Intake Data Collected Using Web-Based and Traditional Nutrition Surveillance Approaches: Comparison and Usability Study
Source: JMIR Public Health Surveill. 2021 Apr 7;7(4):e22759. doi: 10.2196/22759 (PMC8060863; doi:10.2196/22759)
Supplement: Multimedia Appendix 4 [file publichealth_v7i4e22759_app4.docx]

Multimedia Appendix 4: Food group intakes (grams) of adequate reporters from the Foodbook24 Web-based study (2016) and the National Adult Nutrition Survey (2011)

| **Food group** | | | **FB24 Median^o^** | | **FB24 IQR^p^** | | **NANS Median^q^** | | **NANS IQR^r^** |
| --- | --- | --- | --- | --- | --- | --- | --- | --- | --- |
| **Bread, cereals, rice and pasta** | | |  |  | | | |  |  |
| Other breads (e.g. linseed bread) | | | 0 | (0-28) | | | | 0 | (0-20) |
| Other breakfast cereals (e.g. porridge) | | | 0 | (0-208) | | | | 0 | (0-40) |
| Rice & pasta, flours. grains & starch | | | 0 | (0-86.5) | | | | 0 | (0-55.3125) |
| Ready to eat breakfast cereals | | | 0 | (0-30) | | | | 15 | (0-38.0) |
| White sliced bread & rolls | | | 0 | (0-0) | | | | 41.13 | (11.1875-86.125) |
| Wholemeal & brown bread & rolls | | | 42.38 | (0-91.8704) | | | | 42.5 | (0-88.8125) |
| **Beverages** | | |  |  | | | |  |  |
| Alcoholic beverages | | | 0 | (0-250) | | | | 78.38 | (0-447.812) |
| Coffees | | | 125 | (0-295) | | | | 0 | (0-172.688) |
| Teas | | | 375 | (0-715) | | | | 412.5 | (130-666.188) |
| Water | | | 284 | (0-903.322) | | | | 351.25 | (75-784.688) |
| Carbonated beverages | | | 0 | (0-0) | | | | 0 | (0-97.0625) |
| Diet carbonated beverages | | | 0 | (0-0) | | | | 0 | (0-0) |
| **Dairy** | | |  |  | | | |  |  |
| Cheeses | | | 5 | (0-15) | | | | 10 | (0-21.25) |
| Butter (over 80% fat) | | | 4.5 | (0-15) | | | | 0 | (0-4) |
| Whole milk | | | 0 | (0-0) | | | | 37.5 | (0-169.25) |
| Low fat spreads (under 40% fat) | | | 0 | (0-0) | | | | 0 | (0-2.3125) |
| Low fat, skimmed & fortified milks | | | 0 | (0-50) | | | | 1.25 | (0-159.125) |
| Other milks & milk-based beverages | | | 0 | (0-0) | | | | 0 | (0-0) |
| Yoghurts | | | 0 | (0-47) | | | | 0 | (0-56.5625) |
| **Fruit and Vegetables** | | |  |  | | | |  |  |
| Bananas | | | 0 | (0-58) | | | | 0 | (0-49.8125) |
| Citrus fruits | | | 0 | (0-3.53279) | | | | 0 | (0-0) |
| Green vegetables | | | 0 | (0-12.5) | | | | 0 | (0-22.5) |
| Other fruits (e.g. berries, apples etc.) | | | 98.04 | (0-176.846) | | | | 25 | (0-83.25) |
| Other vegetables | | | 28.87 | (0-65.043) | | | | 17.25 | (0-40.0625) |
| Vegetable & pulse dishes | | | 11.57 | (0-42.5) | | | | 0 | (0-25) |
| Potatoes (boiled/baked/mashed) | | | 0 | (0-80) | | | | 60 | (14.6875-116.438) |
| **Meat, eggs and fish** | | |  |  | | | |  |  |
| Beef & Veal | | | 0 | (0-0) | | | | 0 | (0-33.75) |
| Beef & veal dishes | | | 0 | (0-0) | | | | 0 | (0-62.25) |
| Bacon & ham | | | 0 | (0-6.53519) | | | | 15 | (3.25-32.5) |
| Chicken, turkey & game | | | 0 | (0-50.5) | | | | 18.63 | (0-47.8125) |
| Poultry & game dishes | | | 0 | (0-0) | | | | 0 | (0-20.8125) |
| Eggs & egg dishes | | | 0 | (0-57) | | | | 10 | (0-30) |
| Fish & fish products | | | 0 | (0-44) | | | | 0 | (0-39) |
| Fish dishes | | | 0 | (0-0) | | | | 0 | (0-0) |
| Lamb | | | 0 | (0-0) | | | | 0 | (0-0) |
| Lamb, pork & bacon dishes | | | 0 | (0-0) | | | | 0 | (0-0) |
| Meat products | | | 0 | (0-0) | | | | 0 | (0-25) |
| Pork | | | 0 | (0-0) | | | | 0 | (0-0) |
|  |  |  |  | | |  |  |  |  |
| Cakes, pastries & buns | | | 0 | (0-34.2602) | | | | 0 | (0-30) |
| Biscuits including crackers | | | 11.48 | (0-34) | | | | 7.75 | (0-20) |
| Chocolate confectionary | | | 0 | (0-23.5) | | | | 3.75 | (0-16.0625) |
| Ice-creams | | | 0 | (0-0) | | | | 0 | (0-0) |
| Non-chocolate confectionary | | | 0 | (0-0) | | | | 0 | (0-0) |
| Savoury snacks | | | 0 | (0-5) | | | | 0 | (0-9.25) |
| **Soups, sauces and miscellaneous** | | |  |  | | | |  |  |
| Nuts & seeds, herbs & spices | | | 0 | (0-5.65871) | | | | 0 | (0-0.0625) |
| Soups, sauces & miscellaneous foods | | | 14 | (0-49.0734) | | | | 34.5 | (8.25-90) |

o Median intake of food group intake reported in the Foodbook24 Web-based study

p Interquartile range (IQR) of food group intakes reported in the Foodbook24 Web-based survey

q Median intake of food group intakes reported in the National Adult Nutrition Survey in Ireland

r Interquartile range (IQR) of food group intakes reported in the National Adult Nutrition Survey in Ireland.
